# cGAS Senses Human Cytomegalovirus and Induces Type I Interferon Responses in Human Monocyte-Derived Cells

**DOI:** 10.1371/journal.ppat.1005546

**Published:** 2016-04-08

**Authors:** Jennifer Paijo, Marius Döring, Julia Spanier, Elena Grabski, Mohammed Nooruzzaman, Tobias Schmidt, Gregor Witte, Martin Messerle, Veit Hornung, Volkhard Kaever, Ulrich Kalinke

**Affiliations:** 1 Institute for Experimental Infection Research, TWINCORE, Centre for Experimental and Clinical Infection Research, a joint venture between the Helmholtz Centre for Infection Research and the Hannover Medical School, Hannover, Germany; 2 Institute for Molecular Medicine, University Hospital, University of Bonn, Bonn, Germany; 3 Gene Center and Department of Biochemistry, Ludwig-Maximilians-Universität München, Munich, Germany; 4 Institute of Virology, Hannover Medical School, Hannover, Germany; 5 Research Core Unit Metabolomics, Hannover Medical School, Hannover, Germany; Emory Vaccine Center, UNITED STATES

## Abstract

Human cytomegalovirus (HCMV) infections of healthy individuals are mostly unnoticed and result in viral latency. However, HCMV can also cause devastating disease, e.g., upon reactivation in immunocompromised patients. Yet, little is known about human immune cell sensing of DNA-encoded HCMV. Recent studies indicated that during viral infection the cyclic GMP/AMP synthase (cGAS) senses cytosolic DNA and catalyzes formation of the cyclic di-nucleotide cGAMP, which triggers stimulator of interferon genes (STING) and thus induces antiviral type I interferon (IFN-I) responses. We found that plasmacytoid dendritic cells (pDC) as well as monocyte-derived DC and macrophages constitutively expressed cGAS and STING. HCMV infection further induced cGAS, whereas STING expression was only moderately affected. Although pDC expressed particularly high levels of cGAS, and the cGAS/STING axis was functional down-stream of STING, as indicated by IFN-I induction upon synthetic cGAMP treatment, pDC were not susceptible to HCMV infection and mounted IFN-I responses in a TLR9-dependent manner. Conversely, HCMV infected monocyte-derived cells synthesized abundant cGAMP levels that preceded IFN-I production and that correlated with the extent of infection. CRISPR/Cas9- or siRNA-mediated cGAS ablation in monocytic THP-1 cells and primary monocyte-derived cells, respectively, impeded induction of IFN-I responses following HCMV infection. Thus, cGAS is a key sensor of HCMV for IFN-I induction in primary human monocyte-derived DC and macrophages.

## Author Summary

Human cytomegalovirus (HCMV) has been shown to induce type I interferon (IFN-I) responses in myeloid cells such as plasmacytoid dendritic cells (pDC). Although these cells were reported to sense the viral DNA genome in a Toll-like receptor (TLR)-dependent manner, previous studies showed that individuals displaying a hypo-functional TLR axis do not show increased incidence of HCMV infection. This implies that in addition to TLR other sensing mechanisms played a role. Recently cytosolic cyclic GMP/AMP synthase (cGAS) was reported to sense cytosolic DNA and subsequently to induce IFN-I via the production of the cyclic di-nucleotide cGAMP, which activates the stimulator of interferon genes (STING). However, the role of cGAS in recognition of HCMV by human immune cells has not been addressed, yet. In this study we found that pDC as well as monocyte-derived dendritic cells (DC) and macrophages express cGAS and STING. Although pDC expressed particularly high levels of cGAS, they sensed HCMV via TLR9. In contrast, monocyte-derived DC and macrophages sensed the virus in a cGAS-dependent manner. Thus, different innate immune cell subsets deploy different recognition platforms for the detection of the same pathogen. Such information will help to better understand the complex host pathogen balance in humans.

## Introduction

Human cytomegalovirus (HCMV) is a highly host-adapted, opportunistic β-herpesvirus that copes amazingly well with the host’s immune response due to a plethora of different evasion mechanisms [1,2]. These strategies allow HCMV to establish latency and to silently spread to naïve individuals. Currently 60–100% of the world population is latently infected with HCMV [3]. In immunocompromised hosts, e.g., transplant recipients, HCMV reactivation may cause serious disease, while congenital infection can lead to abortion or dramatic disabilities in the infant, such as deafness and mental retardation [4,5].

As a first line of antiviral defense, innate immune cells express cytokines that activate and recruit innate as well as adaptive immune cells. One of the earliest and most prominent families of antiviral cytokines are the type I interferons (IFN-I). IFN-I in humans comprise 13 functional IFN-α genes that encode intronless mRNAs, two of which (IFN-α1 and IFN-α13) presumably were derived from gene duplication and encode identical protein sequences, and one single IFN-β [6–8]. IFN-I facilitate immune responses against viruses by inducing an antiviral state in host cells and orchestrating innate as well as adaptive immune responses [9,10]. They are induced upon sensing of pathogen associated molecular patterns by pattern recognition receptors (PRR) such as Toll-like receptors (TLR), RIG-I like helicases (RLH), and more recently identified intracellular DNA sensors [11,12]. In CMV infection IFN-I play a key role as indicated by IFN-I receptor (IFNAR) deficient mice that show highly increased sensitivity to infection with murine CMV (MCMV) [13,14]. The evolution of multiple CMV evasion mechanisms targeting IFN-I induction and IFNAR signaling further underscores the significance of the IFN-I system [15–19]. In MCMV infection, typically two waves of IFN-I expression are detected [20]. While the cellular source and the underlying recognition platform of the first IFN-I wave are still poorly defined, it was shown previously that the second IFN-I wave is primarily contributed by plasmacytoid dendritic cells (pDC) [21,22], which are known for their extraordinary IFN-I production capacity [23,24]. This IFN-I response is highly dependent on endosomally-located TLR9 [25], which recognizes double-stranded hypomethylated CpG-rich DNA and signals via the adaptor molecule MyD88 [26]. Mice deficient for components of the TLR9 axis showed increased mortality upon MCMV infection [27]. Reminiscent of conditions in mice, also HCMV-stimulated human pDC mount substantial IFN-α responses, while pDC are largely resistant to HCMV infection when compared with either CD11c^+^ DC or freshly isolated monocytes [28,29]. Treatment of human pDC with TLR7/9 inhibitory CpG oligodinucleotides (ODN) abrogated HCMV induced IFN-α responses [30] indicating that also in human pDC TLR play a central role in HCMV recognition. However, patients suffering from primary MyD88 deficiency did not show increased susceptibility to infection with herpesviruses [31,32] questioning the central function of pDC in the pathogenesis of HCMV in humans and suggesting the involvement of other cells and recognition platforms in the production of protective IFN-I.

In human fibroblasts several intracellular dsDNA receptors, such as DNA-dependent activator of interferon regulatory factors (DAI, also known as ZBP1) [33] and interferon-γ inducible protein 16 (IFI16) [34], have been identified to recognize HCMV [35–37]. Yet, fibroblasts mount only weak IFN-I responses, whereas in addition to pDC other myeloid cells such as monocytes and monocyte-derived macrophages (moMΦ) have been reported to produce abundant IFN-I following stimulation with MCMV or HCMV [38–40]. As myeloid cells are also sites of HCMV latency [41–43] and may contribute to HCMV dissemination [44], their role as sentinels of HCMV infection is of particular interest. Studies with *in vitro* polarized macrophages reported that pro-inflammatory macrophages are less susceptible to HCMV infection than anti-inflammatory macrophages, whereas both cell types can be productively infected [39,45]. Murine macrophages stimulated with HCMV mount IFN-I responses that are dependent on the adaptor protein stimulator of interferon genes (STING) [46,47]. Furthermore, in human macrophages the dsDNA receptor IFI16 that was shown to associate with STING [34] seems to play a role in HCMV sensing [48]. Recently, the cytosolic dsDNA receptor cyclic GMP/AMP synthase (cGAS) was identified to be activated upon DNA binding and to produce the second messenger cyclic 2´-5´/3´-5´ GMP/AMP (cGAMP) [49–52]. cGAMP, that may also spread via gap junctions to bystander cells [53], directly activates STING that then translocates from the endoplasmic reticulum to perinuclear punctate structures in order to mediate IFN-I induction via TANK-binding kinase 1 (TBK1) and interferon regulatory factor 3 (IRF3) [46,47,50,52]. In human foreskin fibroblasts cGAS has been shown not only to be a direct sensor of cytosolic DNA, but also to stabilize IFI16 against proteasomal degradation [54]. Nevertheless, to date in human antigen presenting cell subsets the involvement of cGAS in HCMV recognition and subsequent IFN-I induction remains to be elucidated.

Here we investigated the role of the cGAS/STING axis in HCMV induced IFN-I responses of primary human pDC as well as of monocyte-derived DC (moDC), GM-CSF MΦ, and M-CSF MΦ. We found that cGAS and STING were expressed by these cell subsets to varying degrees. Furthermore, HCMV induced intracellular cGAMP formation was detected in monocyte-derived DC and MΦ, but not in pDC. Finally, an essential role for cGAS in HCMV recognition and subsequent IFN-I induction was confirmed by siRNA mediated cGAS knock-down in primary human monocyte-derived cells.

## Results

### HCMV infection enhances cGAS expression in pDC and monocyte-derived cells, while STING expression is differentially regulated

**Fig 1:**
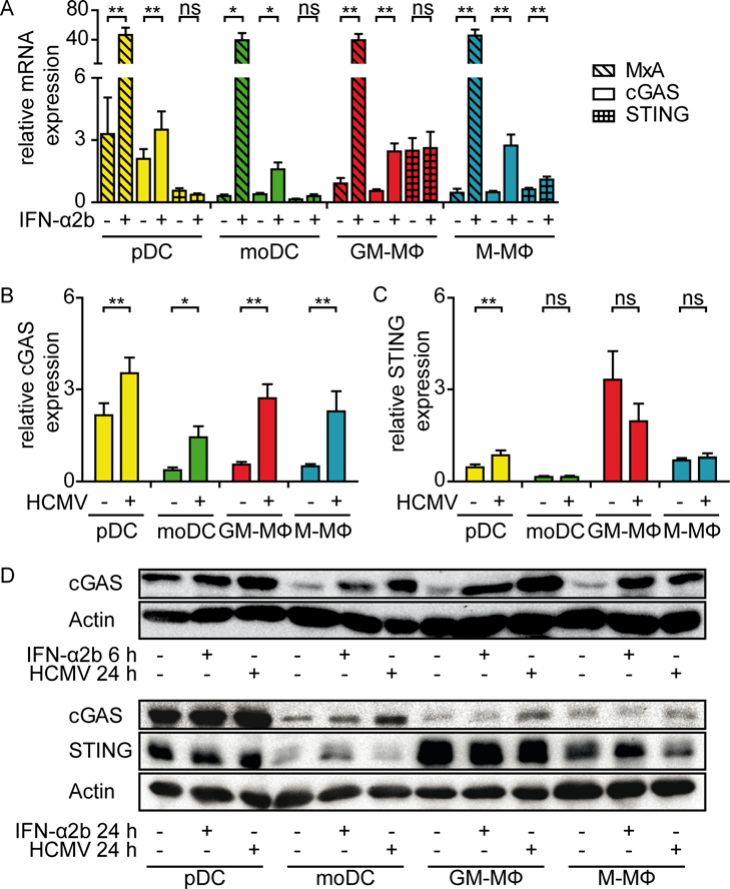
pDC and monocyte-derived cells show constitutive cGAS expression that is enhanced upon IFN-α or HCMV stimulation. Primary human pDC (yellow bars), moDC (green bars), GM-CSF MΦ (GM-MΦ, red bars), and M-CSF MΦ (M-MΦ, blue bars) were (A) stimulated for 6 h with recombinant IFN-α2b and analyzed for relative MxA, cGAS, and STING mRNA expression. Furthermore, the cells were infected with HCMV at MOI 3 for 24 h and analyzed for (B) cGAS and (C) STING mRNA expression relative to HPRT1 mRNA by qPCR. (D) Protein levels of cGAS and STING upon HCMV infection for 24 h or recombinant IFN-α2b treatment for 6 h (upper panel) or 24 h (lower panel) were determined by western blot analysis, while actin was used as loading control. Mean ± SEM of 5–14 (A), 5–15 (B, C) or representative for 3–4 (D) different donors. ns = not significant, *: p ≤ 0.032, **: p ≤ 0.0078 one-tailed Wilcoxon signed rank test.

To address the role of the cGAS/STING pathway in HCMV infected primary human immune cells, in a first step we studied cGAS and STING expression of pDC, moDC, GM-CSF MΦ, and M-CSF MΦ. To this end, we isolated primary human pDC and monocytes from PBMC and *in vitro* differentiated moDC, GM-CSF MΦ, and M-CSF MΦ from monocytes **(S1 Fig)**. Freshly isolated as well as 24 h cultivated pDC showed enhanced levels of cGAS mRNA expression, whereas monocyte-derived cells expressed lower amounts of basal cGAS mRNA **(Fig 1A and 1B and S2 Fig)**. On the contrary, STING mRNA expression was abundant in GM-CSF MΦ, while pDC and M-CSF MΦ showed intermediate, and moDC rather low level expression **(Fig 1A and 1C)**. Upon stimulation for 6 h with recombinant IFN-α2b, cGAS mRNA expression as well as the classical interferon stimulated gene (ISG) MxA were significantly induced in all cell subsets analyzed, whereas STING mRNA expression remained overall stable and was only moderately enhanced in M-CSF MΦ **(Fig 1A)**. Similarly, HCMV (TB40/E) treatment for 24 h induced cGAS mRNA in all cell types analyzed to comparable levels as observed upon IFN-α2b stimulation **(compare Fig 1A and 1B)**. In contrast, upon HCMV treatment STING mRNA expression was moderately enhanced only in pDC, whereas it stayed overall unchanged in moDC and M-CSF MΦ and showed the tendency of even reduced expression in GM-CSF MΦ **(Fig 1C)**. Western blot analysis confirmed cGAS induction upon IFN-α2b or HCMV stimulation, although minor donor-dependent variabilities were observed **(Fig 1D)**. In contrast, moderate STING induction was detected only upon IFN-α2b stimulation for 24 h in moDC and M-CSF MΦ, but not upon HCMV treatment **(Fig 1D)**. Thus, pDC, moDC, GM-CSF MΦ, and M-CSF MΦ expressed the main components of the cGAS/STING axis, although expression levels differed between the myeloid cell subsets. While pDC expressed particularly high levels of inducible cGAS, moDC expressed very low levels of STING.

### Upon HCMV infection, pDC produce larger amounts of IFN-α compared with M-CSF MΦ, GM-CSF MΦ, and moDC

**Fig 2:**
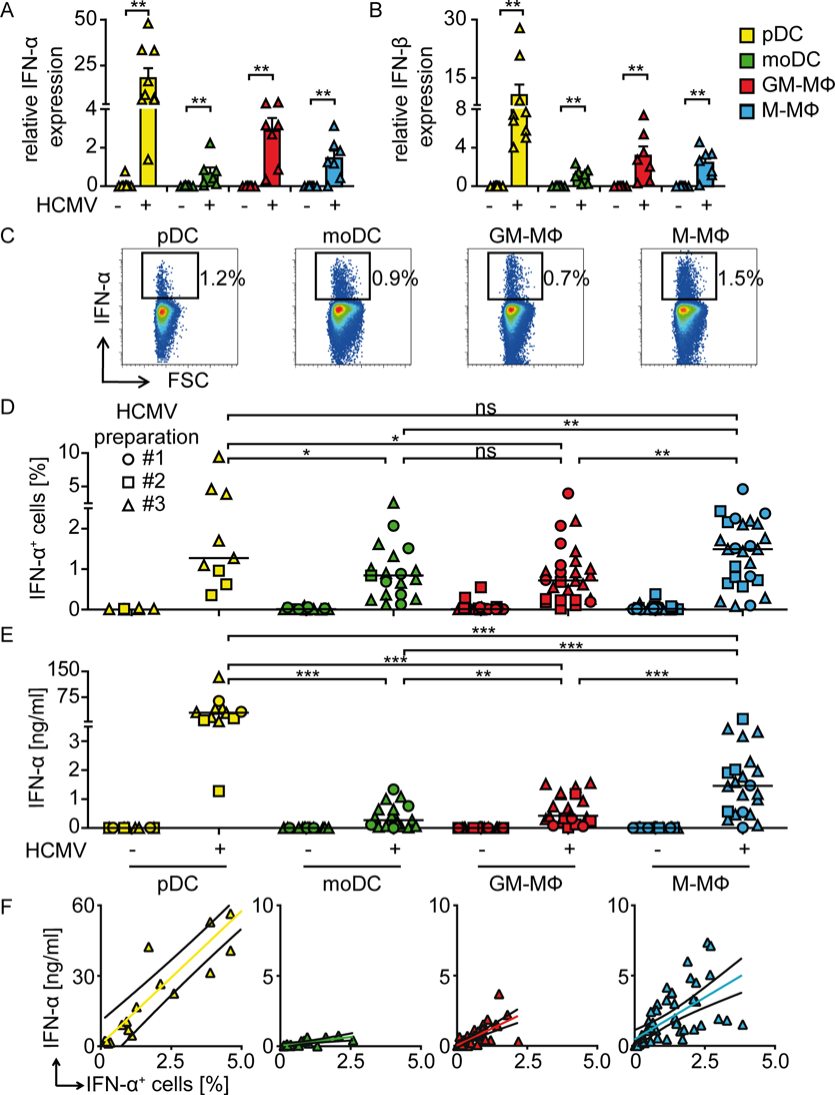
HCMV infection induces IFN-I responses in pDC as well as monocyte-derived DC and MΦ. pDC, moDC, GM-CSF MΦ, and M-CSF MΦ were infected with HCMV at MOI 3 and 24 hours post infection (hpi) (A) IFN-α and (B) IFN-β mRNA expression was determined relative to HPRT1 mRNA by qPCR (mean ± SEM of 7–9 different donors). (C, D) IFN-α expressing cells were analyzed by flow cytometry and (E) the IFN-α content in cell-free supernatants was determined by an ELISA method upon infection with three independently produced HCMV preparations (#1, #2, and #3) at MOI 3. (F) Upon HCMV infection with varying MOI between 0.1 to 30 IFN-α concentrations in cell-free supernatants (y-axis) were blotted against percentages of IFN-α^+^ cells (x-axis) of the same culture (Values from 12–28 different donors. Black line: confidence interval of 95%). Median of 9–26 (D) and 12–23 (E) different donors. ns = not significant, *: p ≤ 0.031, **: p ≤ 0.0078, ***: p ≤ 0.0002 one-tailed paired Wilcoxon signed rank test was used for statistical analyses between monocyte-derived cell subsets, because the subsets were derived from monocytes of the same donors. In contrast, pDC were derived from different donors. Therefore, statistical analysis between pDC and one of the monocyte-derived cell subsets was performed using one-tailed unpaired Mann-Whitney test.

To study IFN-I expression by pDC, moDC, GM-CSF MΦ, and M-CSF MΦ, cells were infected with HCMV at MOI 3 for 24 h. qPCR analysis revealed significant IFN-α and IFN-β induction in all HCMV treated cell subsets, which was particularly strong in pDC **(Fig 2A and 2B)**. Quantification of IFN-α expression on the single cell level indicated that HCMV treated pDC and M-CSF MΦ comprised higher percentages of IFN-α positive cells (1.2 and 1.5%, respectively) than moDC and GM-CSF MΦ (0.9 and 0.7%, respectively) **(Fig 2C and 2D)**. As expected, also cell-free culture supernatants contained substantial amounts of IFN-α that were approximately 10 to 50-fold higher in supernatants from pDC compared with the other cell types. However, low to robust IFN-α levels were detected in supernatants of moDC, GM-CSF MΦ, and M-CSF MΦ cultures **(Fig 2E)**. Furthermore, we found that upon HCMV stimulation the amount of IFN-α secreted per IFN-α^+^ cell was particularly high in pDC, while GM-CSF MΦ as well as M-CSF MΦ produced intermediate and moDC particularly small quantities **(Fig 2F)**. Thus, upon HCMV infection pDC mounted the most abundant IFN-I responses, whereas the two analyzed MΦ subsets also showed high IFN-I expression upon HCMV infection. Surprisingly, HCMV infected moDC showed especially low IFN-α production when compared with the other cell subsets, as determined by the absolute quantities detected in the cell-free supernatant as well as the amount of IFN-α produced per IFN-α^+^ cell.

### HCMV infection of monocyte-derived DC and MΦ, but not of pDC, induces formation of cytosolic cGAMP that precedes IFN-α secretion

**Fig 3:**
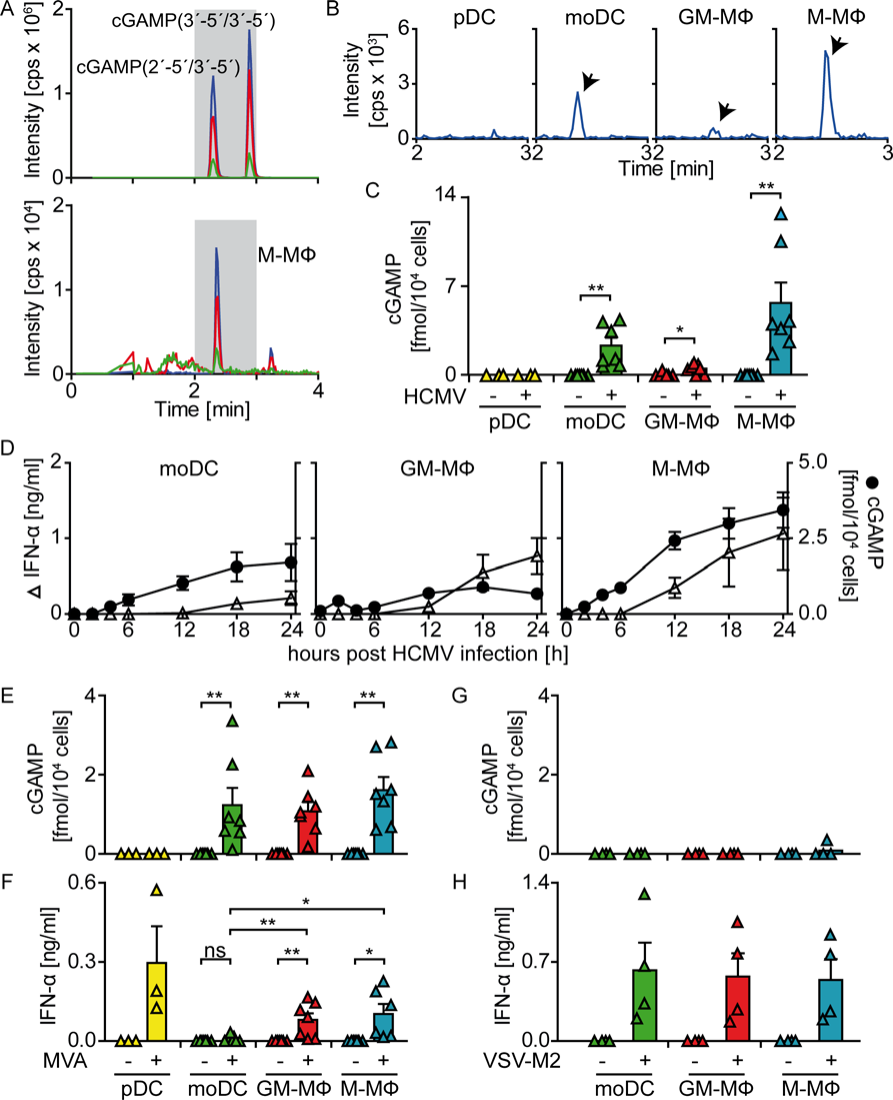
HCMV infection induces cGAMP formation in monocyte-derived DC and MΦ, but not in pDC. cGAMP synthesis was analyzed and quantified using a HPLC-MS/MS method. Chromatograms (blue line: quantifier, green/red lines: identifiers) of (A) synthetic cGAS-derived cGAMP (2´-5´/3´-5´) and bacterial cGAMP (3´-5´/3´-5´) (upper panel) or lysed, HCMV stimulated M-CSF MΦ (lower panel) as well as (B) lysates of 24 h HCMV infected pDC, moDC, GM-CSF MΦ, and M-CSF MΦ (enlarged visualization of grey area shown in (A)). (C) Quantification of the detected cGAMP shown in (B). (D) cGAMP (filled circles) synthesis and IFN-α contents in cell-free supernatants (open triangles) were monitored in unstimulated (0 h) moDC, GM-CSF MΦ, and M-CSF MΦ or at indicated time points after HCMV treatment. pDC, moDC, GM-CSF MΦ, and M-CSF MΦ were infected with MVA at MOI 1 and 24 hpi (E) cGAMP synthesis as well as (F) IFN-α contents of cell-free supernatants were quantified. moDC, GM-CSF MΦ, and M-CSF MΦ were infected with VSV-M2 at MOI 1 and 24 hpi (G) cGAMP synthesis as well as (H) IFN-α contents of cell-free supernatants were quantified. Mean ± SEM of 4–7 (C), 4 (D), 3–7 (E, F), or 4 (G, H) different donors. ns = not significant, *: p ≤ 0.032, **: p ≤ 0.0078 one-tailed Wilcoxon signed rank test.

To next address whether cGAS played a role in the recognition of HCMV by pDC, moDC, GM-CSF MΦ, and M-CSF MΦ, we aimed to determine the product of cGAS activation, cGAMP. This was done by a HPLC-MS/MS method that on the basis of different retention times allowed discrimination of cGAS-derived 2´-5´/3´-5´ cGAMP and of other cyclic di-nucleotides such as the bacterial-derived 3´-5´/3´-5´ cGAMP (**Fig 3A)**. Interestingly, despite pDC expressed abundant levels of cGAS and STING **(see Fig 1)**, in lysates of HCMV treated cells no cGAMP was detected **(Fig 3B and 3C)**. Analysis of lysates of HCMV stimulated monocyte-derived cells revealed that M-CSF MΦ contained particularly high cGAMP levels **(Fig 3B and 3C)**, whereas in moDC and GM-CSF MΦ intermediate to low cGAMP levels were detected **(Fig 3B and 3C)**. For the determination of the kinetics of cGAMP formation, monocyte-derived cells were analyzed at different time points after HCMV infection. These experiments revealed that already 2–4 hpi intracellular cGAMP was detected that increased until 24 hpi **(Fig 3D)**. Thus, HCMV induced cGAMP responses of monocyte-derived cells preceded IFN-α secretion, which was first detectable in cell culture supernatants at 12 hpi **(Fig 3D)**. Notably, although at 24 hpi moDC showed higher cGAMP responses than GM-CSF MΦ, moDC secreted significantly less IFN-α than GM-CSF MΦ **(Figs 3D and 2E)**.

Furthermore, cells were stimulated with modified vaccinia virus Ankara (MVA), a highly attenuated vaccinia virus strain, which is encoded by a DNA genome and in murine conventional DC was shown to be sensed in a cGAS-dependent manner [55]. MVA stimulation did not induce cGAMP synthesis in pDC **(Fig 3E)**, whereas IFN-α was detectable in the supernatant **(Fig 3F)**. In contrast, MVA treatment induced cGAMP responses in all monocyte-derived cell subsets tested **(Fig 3E)**. Although overall similar cGAMP levels were detected, the IFN-α levels produced by GM-CSF MΦ and M-CSF MΦ were higher than those of moDC upon MVA stimulation **(Fig 3F)**. As another control, monocyte-derived cells were also treated with RNA encoded VSV-M2 (a vesicular stomatitis virus strain carrying a mutation in the M protein), which is sensed in murine macrophages and conventional DC in a RLH-dependent manner [56,57]. In these experiments cGAMP was not detected in any of the tested cell subsets **(Fig 3G)**, while all three monocyte-derived cell subsets mounted abundant IFN-α responses of similar magnitude **(Fig 3H)**. Thus monocyte-derived cells infected with RNA encoded VSV-M2 did not show cGAMP formation, whereas treatment with DNA encoded HCMV or MVA indeed activated cGAS to synthesize cGAMP.

**Fig 4:**
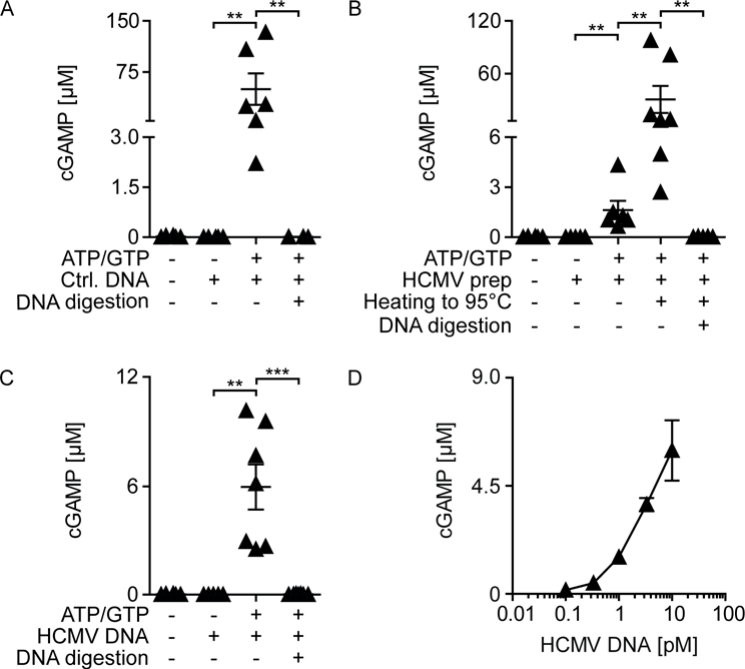
HCMV-derived genomic DNA efficiently activates recombinant human cGAS. Recombinant human cGAS was incubated for 2 h in the presence or absence of ATP and GTP with (A) a 50 bp long control dsDNA, (B) purified HCMV containing approximately 6.5 x 10^6^ infectious virus particles, or purified HCMV treated for 10 minutes at 95°C, or (C, D) genomic DNA isolated from purified HCMV. All samples were tested with and without DNA digestion prior to incubation with recombinant cGAS. cGAMP formation was quantified using a HPLC-MS/MS method. Mean ± SEM of 5–6 (A) and 5–7 (B, C, D) data points from 3 independent experiments. **: p ≤ 0.005, ***: p ≤ 0.0003 one-tailed Mann-Whitney test.

To further verify that in monocyte-derived cells cGAS can be activated by HCMV-derived viral DNA, we analyzed cGAMP synthesis by recombinant human cGAS in a cell-free system. As shown previously, cGAMP was synthesized from ATP and GTP upon activation of recombinant cGAS by a 50 bp control dsDNA **(Fig 4A)** [58]. While in HCMV preparations no cGAMP was detected **(Fig 4B and S3 Fig)**, incubation of cGAS in the presence of ATP and GTP together with HCMV resulted in cGAMP formation at moderate but significant levels **(Fig 4B)**. However, if HCMV preparations were subjected to DNA digestion no cGAMP formation was detected **(S3 Fig)**. In contrast, viral particles that were disrupted by heat treatment induced significantly enhanced cGAMP formation, whereas DNA digestion following heat treatment again inhibited cGAMP synthesis **(Fig 4B and S3 Fig).** These experiments indicated that in heat treated HCMV preparations DNA was the cGAS activating component. To next address whether the viral DNA genome was able to trigger cGAS, we purified the viral genome from HCMV preparations, quantified the viral DNA by qPCR and incubated recombinant cGAS with 10 pM of this DNA. Indeed, under such conditions significant cGAMP formation was detected, whereas DNA digestion again inhibited the effect **(Fig 4C).** In experiments with decreasing amounts of viral DNA, 0.3 pM of viral DNA still activated cGAS **(Fig 4D)**. Thus, the data obtained in a cell-free system indicated that minute quantities of viral DNA indeed were able to activate human cGAS.

### Although cGAMP treated pDC produce IFN-I, they recognize HCMV in a TLR9-dependent manner

**Fig 5:**
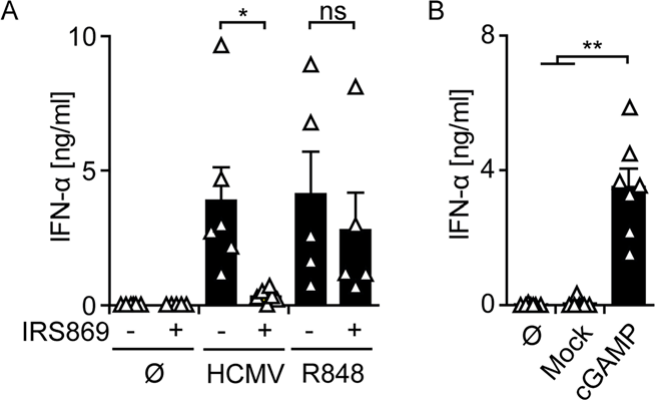
pDC are stimulated by cGAMP to mount IFN-I responses, whereas they sense HCMV in a TLR9-dependent manner. (A) IFN-α responses of pDC were monitored by an ELISA method in supernatants of untreated pDC (Ø), and pDC stimulated with HCMV at MOI 3, or the TLR7/8 agonist R848, in the presence or absence of the TLR9 inhibitory oligonucleotide IRS869. (B) pDC were left untreated (Ø), or mock transfected, or transfected with synthetic cGAMP, and IFN-α expression was monitored by an ELISA method. Mean ± SEM of 5–6 (A) and 7 (B) different donors. ns = not significant, *: p ≤ 0.016, **: p ≤ 0.0078 one-tailed Wilcoxon signed rank test.

As pDC showed no activation of cGAS-dependent cGAMP synthesis upon HCMV stimulation and previous studies suggested that endosomal TLR mediated HCMV recognition in pDC [30], we aimed at specifying the involvement of TLR9 in HCMV sensing. Indeed, HCMV induced IFN-α responses of pDC were significantly impaired by the addition of the TLR9 specific antagonistic oligonucleotide IRS869, whereas IFN responses induced by the TLR7/8 agonist R848 were not inhibited **(Fig 5A)**. This indicated that pDC sensed HCMV primarily in a TLR9-dependent manner to mount IFN-I responses. However, since pDC expressed abundant levels of cGAS and STING **(see Fig 1)**, we next addressed whether the cGAS/STING axis was functional. Notably, transfection of pDC with synthetic cGAMP induced robust IFN-α production **(Fig 5B)** implying an effective signaling transduction cascade downstream of STING.

### HCMV infected monocyte-derived DC and MΦ mount IFN-α responses in a cGAS-dependent manner

**Fig 6:**
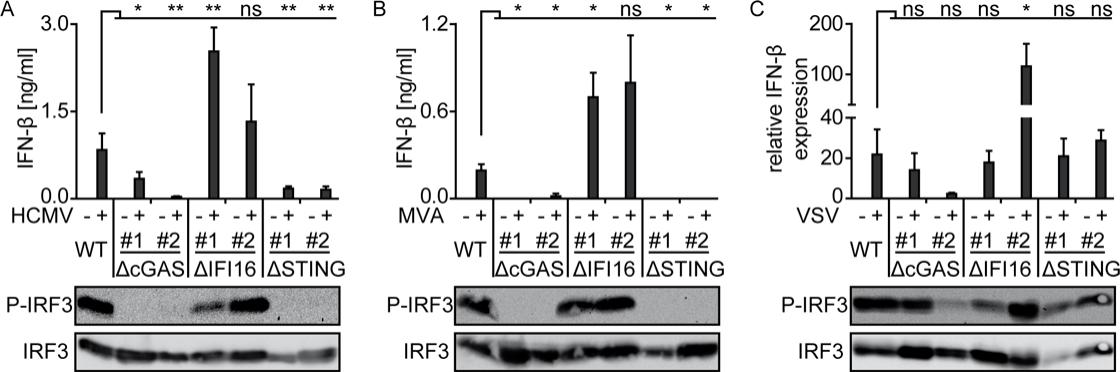
Upon HCMV stimulation PMA-matured THP-1 cells mount IFN-β responses in a cGAS-dependent manner. WT (wild-type), and 2 clones (#1 and #2) of cGAS, IFI16, or STING deficient THP-1 cells differentiated with PMA for 3 days were stimulated with (A) HCMV at MOI 10, (B) MVA at MOI 1, or (C) VSV at MOI 1 for 24 h. Cell-free supernatant was tested for IFN-β by an ELISA method (A, B), or cell lysates were tested for IFN-β mRNA expression relative to HPRT1 mRNA by qPCR (C). Lysates of virus infected cells were analyzed for phosphorylated IRF3 (P-IRF3) and IRF3 by western blot (A, B, C). Mean ± SEM of 3–6 (A), 3 (B), and 4–5 (C) data points from 3 (A, C) and 2 (B) independent experiments. *: p ≤ 0.05, **: p ≤ 0.0076 one-tailed Mann-Whitney test.

We next addressed the role of cGAS in HCMV mediated IFN-I induction in myeloid cells. As the DNA sensor IFI16 has been reported to be involved in HCMV sensing and IFN-I production of macrophages [48] and to cooperate with cGAS for DNA sensing in fibroblasts [54], we also studied the role of IFI16 in HCMV induced IFN-I responses. We analyzed monocytic THP-1 cells in which cGAS, IFI16, or STING was deleted by CRISPR/Cas9 technology **(S4 Fig)** [59]. These cells were used either undifferentiated **(S5 Fig)** or upon differentiation by incubation with PMA **(Fig 6)**. Unlike WT THP-1 cells, HCMV treated cGAS or STING deficient THP-1 cells showed significantly impaired IFN-β production **(Fig 6A and S5A Fig)**. In contrast, HCMV treated IFI16 deficient THP-1 cells mounted abundant IFN-β responses that were even moderately enhanced when compared with WT THP-1 cells **(Fig 6A and S5A Fig)**. Western blot analysis of phosphorylated IRF3 (P-IRF3), which is an indicator of downstream signaling of STING, revealed the presence of P-IRF3 only in HCMV treated WT and IFI16 deficient THP-1 cells, whereas it was not detected in cGAS and STING deficient THP-1 cells **(Fig 6A and S5A Fig)** as well as in unstimulated cells **(S5D and S5E Fig)**. Similarly, treatment with DNA encoded MVA triggered IFN-β responses only in WT and IFI16 deficient THP-1 cells, whereas upon cGAS or STING ablation no responses were detected **(Fig 6B and S5B Fig)**. Again only WT and IFI16 deficient THP-1 cells, but not cGAS or STING deficient THP-1 cells, showed P-IRF3 induction upon MVA treatment **(Fig 6B and S5B Fig)**. In contrast, infection with RNA encoded VSV induced IFN-β mRNA expression as well as P-IRF3 in all tested THP-1 variants **(Fig 6C and S5C Fig)**. Interestingly, IFN-β mRNA expression and IRF3 phosphorylation of one cGAS deficient clone were slightly decreased compared with WT THP-1 cells, whereas one IFI16 deficient clone showed enhanced IFN-β responses **(Fig 6C),** which might be explained by off-target effects. Since here we stimulated THP-1 cells with a VSV variant that carried a functional M protein, instead of VSV-M2, as done in experiments shown in Fig 3H, no IFN-β protein secretion was detected due to M protein mediated inhibition [60].

**Fig 7:**
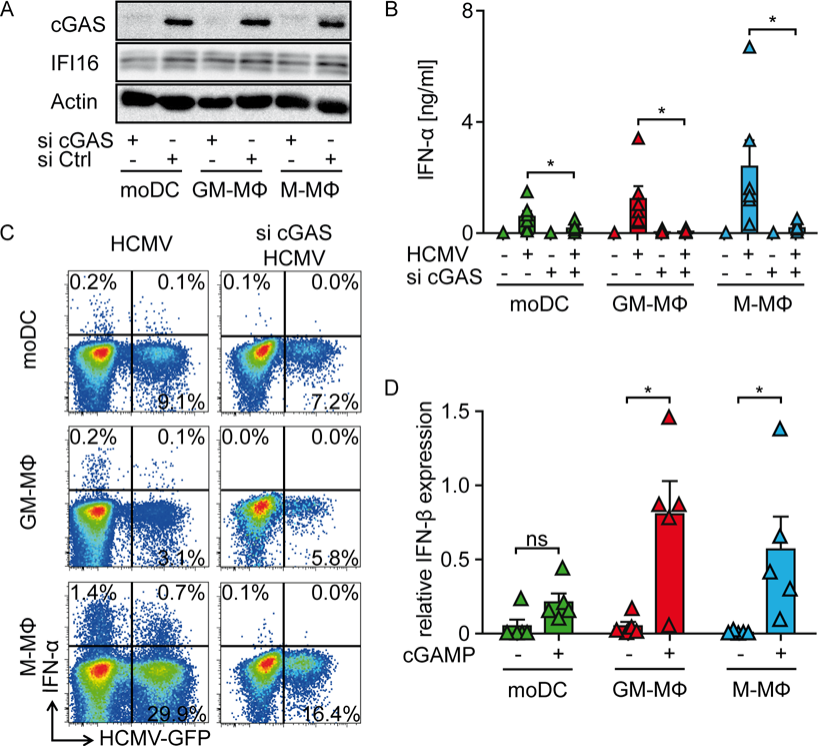
HCMV infected monocyte-derived DC and MΦ mount IFN-α responses in a cGAS-dependent manner. (A) moDC, GM-CSF MΦ, and M-CSF MΦ were transfected with siRNA directed against cGAS or control siRNA and cGAS or IFI16 expression was monitored by western blot analysis, while actin was used as loading control. Untreated or siRNA-mediated cGAS knock-down moDC, GM-CSF MΦ, and M-CSF MΦ were infected with HCMV-GFP at MOI 3 for 24 h and (B) IFN-α contents of cell-free supernatants were monitored by ELISA or (C) IFN-α^+^ and/or HCMV-GFP^+^ cells were analyzed by flow cytometry. (D) cGAS knock-down moDC, GM-CSF MΦ, and M-CSF MΦ were mock transfected or transfected with synthetic cGAMP and analyzed for IFN-β mRNA expression relative to HPRT1 mRNA by qPCR. Mean ± SEM of 6 (B) and 5 (D) or representative for 6 (A, C) different donors from 3 independent experiments. ns = not significant, *: p ≤ 0.04 one-tailed Wilcoxon signed rank test.

To next address, whether HCMV sensing of primary human monocyte-derived cells was similarly dependent on the cGAS/STING axis as detected in THP-1 cells, we applied a Viromer-based transfection method to induce siRNA-mediated knock-down of cGAS in moDC, GM-CSF MΦ, and M-CSF MΦ. Indeed, western blot analysis confirmed efficient and specific cGAS knock-down in siRNA treated monocyte-derived cells, whereas IFI16 expression was not affected **(Fig 7A)**. Upon infection with a HCMV variant expressing GFP under the control of the major immediate early promotor (HCMV-GFP) siRNA-treated monocyte-derived DC and MΦ mounted significantly reduced IFN-α responses **(Fig 7B and 7C)**, while irrespective of siRNA-treatment they showed overall similar percentages of HCMV-GFP^+^ cells **(Fig 7C)**. To ensure that cGAS knock-down would not affect down-stream signaling of STING or stimulation with TLR ligands, we next stimulated cells subjected to siRNA mediated cGAS knock-down with synthetic cGAMP or LPS. cGAS siRNA-treated moDC, GM-CSF MΦ, and M-CSF MΦ that mounted reduced IFN-α responses upon HCMV-GFP treatment were still induced to express IFN-β or TNF-α upon transfection with synthetic cGAMP and stimulation with LPS, respectively **(Fig 7D and S6 Fig)**. Notably, synthetic cGAMP induced IFN-β responses were more abundant in moMΦ than in moDC. Thus, cGAS was not only activated upon HCMV stimulation in monocyte-derived cells, but it was essential to mount abundant IFN-I responses.

### Monocyte-derived cells are more susceptible to HCMV infection than pDC

**Fig 8:**
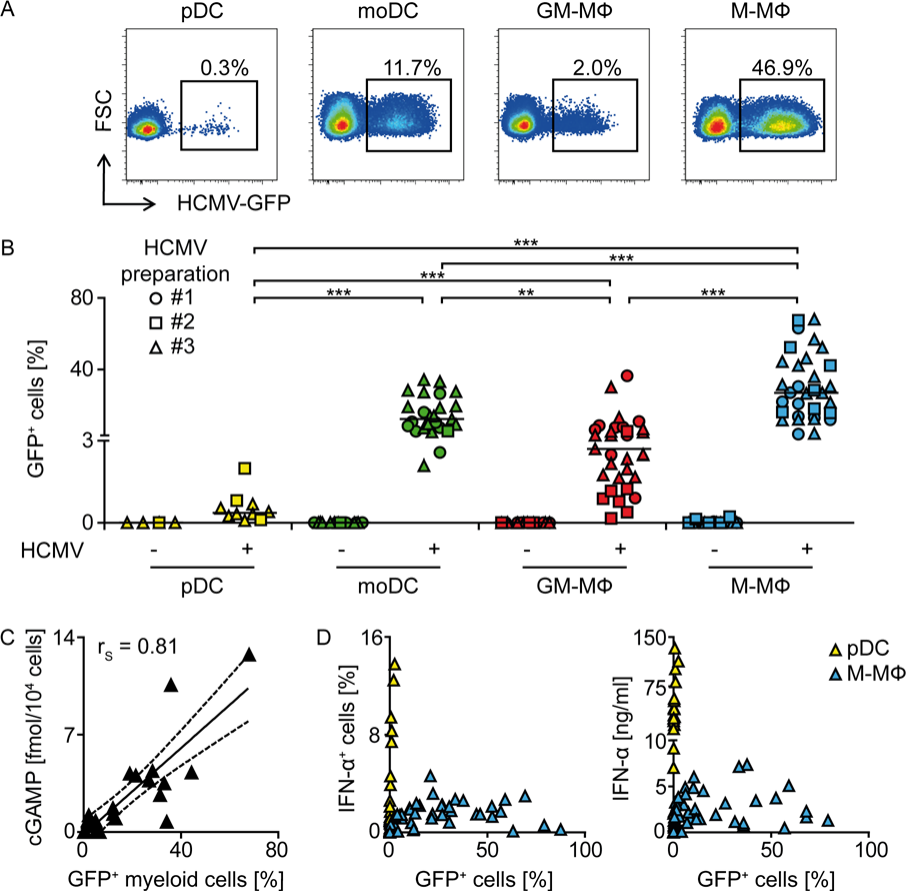
Upon HCMV-GFP infection, GFP^+^ cells are more abundant in monocyte-derived cells than in pDC. (A, B) pDC, moDC, GM-CSF MΦ, and M-CSF MΦ were infected with three independently produced HCMV-GFP preparations (#1, #2, and #3) at MOI 3 for 24 h and percentages of HCMV-GFP^+^ cells were analyzed by flow cytometry. (C) pDC, moDC, GM-CSF MΦ, and M-CSF MΦ were infected with HCMV-GFP and percentages of GFP^+^ cells as well as cGAMP synthesis were determined by FACS analysis and a HPLC-MS/MS method, respectively. Percentages of GFP^+^ cells (x-axis) were blotted against the amount of cGAMP synthesized (y-axis) from the same cultures for all different cell subsets in one blot and the Spearman correlation (r_S_) between the factors was determined. (D) pDC and M-CSF MΦ were infected with HCMV-GFP at varying MOI between 0.1 to 30 and percentages of GFP^+^ cells (x-axis) were blotted against percentages of IFN-α^+^ cells or IFN-α contents of cell-free supernatants of the same cultures (y-axis). Median of 8–30 (B) or values from 3–7 (C) and 14–42 (D) different donors. **: p ≤ 0.0013, ***: p ≤ 0.00031 one-tailed paired Wilcoxon signed rank test was used for statistical analyses between monocyte-derived cell subsets, because the subsets were derived from monocytes of the same donors. In contrast, pDC were derived from different donors. Therefore, statistical analysis between pDC and one of the monocyte-derived cell subsets was performed using one-tailed unpaired Mann-Whitney test.

Previous studies with murine and human immune cells implied that the vulnerability to CMV infection and the support of viral gene expression varied between myeloid cell subsets and that pDC were particularly resistant to infection [28,29,39,40,45]. This led us to hypothesize that the susceptibility to CMV infection was one prerequisite for the engagement of the cytoplasmic cGAS/STING axis in CMV sensing. Indeed, upon HCMV-GFP infection at MOI 3, pDC exhibited the least GFP expression as indicated by 0.3% GFP^+^ cells **(Fig 8A and 8B)**. Analysis of monocyte-derived cells revealed that M-CSF MΦ contained the highest percentages of HCMV-GFP^+^ cells (46.9%), whereas moDC exhibited intermediate and GM-CSF MΦ low amounts of GFP^+^ cells (11.7 and 2.0%, respectively) **(Fig 8A and 8B)**. Notably, cell subsets that showed higher percentages of HCMV-GFP^+^ cells also synthesized enhanced cGAMP levels **(compare Figs 8B and 3C)**. Indeed, upon combined analysis of pDC, moDC, GM-CSF MΦ, and M-CSF MΦ we verified that the percentage of HCMV-GFP^+^ cells correlated with the amount of cGAMP that was formed in myeloid cells **(Fig 8C)**.

To analyze the correlation between infection with HCMV and IFN-α production, we stimulated pDC and M-CSF MΦ with gradually increasing MOI (MOI 0.1–30) of HCMV-GFP. In this setting, the percentage of IFN-α^+^ pDC and the amount of IFN-α secreted into the supernatant increased up to levels of 15% and 130 ng/ml, respectively, whereas the percentage of GFP^+^ cells stayed comparably low **(Fig 8D)**. In contrast, in M-CSF MΦ cultures highly increased percentages of HCMV-GFP^+^ cells were detected **(Fig 8D)**. These correlated with increased percentages of IFN-α^+^ cells as well as enhanced amounts of secreted IFN-α up to approximately 40% GFP^+^ cells, whereas at further enhanced percentages of GFP^+^ cells IFN-α responses waned **(Fig 8D)**. These data indicated that in monocyte-derived M-CSF MΦ, but not pDC, IFN-α responses were the higher the more HCMV-GFP^+^ cells were detected until reaching a certain infection threshold. In conclusion, our data showed that the susceptibility of the different myeloid cell subsets to HCMV infection correlated with the amount of synthesized cGAMP. Abundantly HCMV infected monocyte-derived DC and MΦ showed enhanced cGAMP formation, whereas in pDC, which were highly resistant to infection, cGAMP synthesis was not detected.

## Discussion

The knowledge of diverse mechanisms applying for primary human immune cell sensing of human-specific viruses, such as HCMV, is of major importance to better understand complex virus/host interactions. Here we report that in primary human monocyte-derived DC and MΦ cGAS was essential for HCMV sensing and subsequent IFN-I induction. The degree of cGAS-dependent cGAMP formation correlated with the susceptibility of different monocyte-derived cell subset to HCMV infection. In line with this observation, pDC that were not readily HCMV infected mounted IFN-I responses in a TLR9-dependent manner, although they expressed abundant amounts of cGAS and STING.

Our observation that pDC are triggered by HCMV in a TLR9-dependent manner was based on selective TLR9 inhibition using the IRS869 oligonucleotide [61] and confirmed an earlier study in which a TLR7/9 inhibitory CpG ODN was used [30]. Interestingly, pDC expressed enhanced constitutive cGAS levels compared with monocyte-derived cells, which were significantly induced upon IFN-α2b or HCMV stimulation. A previous study showed that upon *in vitro* cultivation pDC spontaneously expressed low levels of IFN-I [62], which might have accounted for the enhanced basal expression of cGAS in pDC. However, this was not the case, because also freshly isolated pDC expressed high cGAS levels. Therefore, we conclude that already naïve pDC show an inherent interferon pre-activation status that facilitates swift responses to pathogens [63]. It is possible that in human pDC enhanced constitutive cGAS levels further supported this status as suggested by experiments with murine macrophages in which cGAS expression was a prerequisite for normal levels of constitutive ISG expression [64]. Furthermore, pDC constitutively expressed abundant levels of STING that slightly increased upon HCMV stimulation. Transfection of pDC with synthetic cGAMP induced IFN-α responses, indicating that in pDC the cGAS/STING axis downstream of STING was fully functional. Nevertheless, a contribution of the cGAS/STING axis in HCMV sensing and IFN-I induction of pDC was not observed, because upon HCMV stimulation of pDC no intracellular cGAMP was detected. This raises the question why in pDC the abundantly expressed cGAS was not activated upon HCMV stimulation to synthesize cGAMP. One possible explanation is that cGAS activity is reduced in pDC by mechanisms like cGAS glutamylation and/or beclin-1 interaction, which have been reported to reduce cGAS DNA binding and enzymatic activity [65,66]. However, our observations that the degree of HCMV-GFP infection and the extent of cGAMP production correlated in myeloid cells, and that pDC were not efficiently infected, suggested that in pDC the viral genome did not reach the cytoplasm to trigger cGAS, and instead directly entered the endosomal/lysosomal pathway to trigger TLR9. A previous study showed that naïve pDC were triggered by the yellow fever live vaccine YF-17D in a RIG-I-dependent manner, whereas upon contact with YF-17D infected cells IFN-I induction was dependent on endosomally located TLR7 [67]. Thus, it is conceivable that depending on how pDC encounter nucleic acids they might also be triggered in a cGAS-dependent manner. Furthermore, in pDC constitutive expression of cGAS might also be associated with not yet defined functions other than virus sensing. In contrast, constitutive STING expression and the resulting sensitivity to cGAMP might be a mechanism of pDC to directly respond to viruses that carry cGAMP in their virions such as HIV-1, MVA, and MCMV [68,69]. Although we did not detect cGAMP in HCMV preparations, we cannot exclude the presence of cGAMP levels in the virus at concentrations below the detection limit of the HPLC-MS/MS method we used, which still might suffice to further boost pDC stimulation.

To address the role of the cGAS/STING axis in monocyte-derived cells we analyzed cGAS and STING expression in moDC and moMΦ. Interestingly, stimulation with recombinant IFN-α2b and HCMV increased cGAS mRNA expression to a similar extent, although upon HCMV infection IFN-I production of single myeloid cell subsets differed significantly. In contrast, treatment with recombinant IFN-α2b moderately induced STING expression in moDC and M-CSF MΦ, whereas HCMV infection did not induce STING, although under such conditions the cells produced IFN-I. As STING rapidly degrades upon activation [70], it is possible that virus induced IFN-I responses compensated virus induced STING activation and degradation. Thus, these results were compatible with the conclusion that upon HCMV infection of monocyte-derived cells STING was activated, as similarly observed in a previous study with murine bone marrow-derived macrophages that also sensed HCMV in a STING-dependent manner [47]. Furthermore, the detection of cGAMP formation in HCMV stimulated monocyte-derived cells proved the activation of cGAS. Additionally, we confirmed that stimulation with DNA-encoded MVA, which was shown to induce cGAS-dependent responses in murine conventional DC [55], also led to cGAMP formation in primary human monocyte-derived DC and MΦ. Because stimulation of monocyte-derived cells with the RNA-encoded VSV-M2 did not induce cGAMP responses and *in vitro* incubation of recombinant cGAS with HCMV DNA resulted in efficient cGAMP synthesis, we conclude that cGAS was activated by binding to the viral DNA genome. Previous studies showed that macrophages evolved a mechanism to degrade the capsid of HSV-1 to release viral DNA into the cytoplasm and that also HCMV DNA co-localized with the cellular protein IFI16 in the cytoplasm [48]. These data support the hypothesis that in HCMV infected human macrophages the viral DNA genome is released into the cytosol, where it activates cGAS. Additionally, HSV-1 was reported to induce cellular stress and subsequent release of mitochondrial DNA into the cytosol, which enhances cGAS activation [71]. Such a mechanism could similarly augment sensing of HCMV infections in monocyte-derived cells by cGAS.

Furthermore, impaired IFR3 phosphorylation and IFN-β induction in cGAS or STING deficient monocytic THP-1 cells demonstrated that the cGAS/STING axis was required for efficient IFN-I expression upon HCMV infection. Interestingly, IFI16 deficiency did not inhibit HCMV induced IFN-I expression in THP-1 cells. This was in contrast to an earlier study, which showed reduced IFN-I responses in HCMV stimulated THP-1 cells upon shRNA mediated cGAS knock-down [48]. This discrepancy might be explained by the different methods used to ablate IFI16, i.e., CRISPR/Cas9 mediated complete IFI16 knock-out vs. shRNA mediated IFI16 knock-down. Additionally, in human fibroblasts cGAS knock-down was reported to increase IFI16 proteasomal degradation [54]. Nevertheless, in cGAS knock-out THP-1 cells we detected normal IFI16 levels by western blot analysis. Thus, in our experiments IFI16 did not compensate for cGAS deficiency upon HCMV stimulation. In previous studies, IFI16 was identified as an important immune sensor of HCMV in human fibroblasts, however, its activity is inhibited by the HCMV protein pUL83 (also known as pp65) [37]. Since pUL83 is the most abundant tegument protein of HCMV with more than 2000 molecules being integrated into the mature virion [72], inhibition of IFI16 may occur right after HCMV infection without the need of viral gene expression. Thus, IFI16 might be inhibited in HCMV infected macrophages and therefore is unable to compensate cGAS deficiency.

To furthermore analyze the requirement of cGAS for IFN-I expression in primary human immune cells we applied a Viromer-based siRNA delivery approach that efficiently inhibited cGAS translation. We demonstrated that also in primary human monocyte-derived cells IFI16 was still present after siRNA mediated cGAS knock-down. HCMV stimulated monocyte-derived cells showed dramatically reduced IFN-α responses upon cGAS knock-down, confirming that IFI16 did not compensate cGAS deficiency and that cGAS was essential for efficient IFN-I expression in primary human monocyte-derived cells.

Of note, HCMV stimulated moDC and moMΦ produced different levels of cGAMP, which correlated with the percentage of cells supporting HCMV gene expression. Thus, M-CSF MΦ that showed the highest percentage of HCMV infected cells, and also in previous studies have been shown to be particularly vulnerable to HCMV infection [45], produced the highest amount of intracellular cGAMP. Therefore, we hypothesize that the susceptibility to HCMV infection determines the accessibility of the viral genome to cGAS, thus directly affecting the magnitude of cGAMP responses. However, intracellular cGAMP levels did not always correlate with the magnitude of the resulting IFN-α responses. moDC expressed similar amounts of cGAS compared with moMΦ (as shown by western blot and qPCR), and produced higher or similar levels of cGAMP compared with GM-CSF MΦ (as shown by mass spectrometry analysis) upon HCMV or MVA infection; however, moDC produced significantly less IFN-I than moMΦ. Additionally, transfection of synthetic cGAMP into cGAS knock-down monocyte-derived cells induced less IFN-β in moDC than in moMΦ. Importantly, upon recognition of VSV, which is recognized in a cGAS-independent manner, moDC and moMΦ mounted similar IFN-I responses. These experiments suggested that in moDC cGAS-dependent IFN-I induction was limited downstream of cGAMP. The comparably low STING expression detected in moDC may account for this limitation of cGAMP-dependent IFN-I expression.

In conclusion, here we report that human pDC as well as monocyte-derived cells abundantly express cGAS and STING. Although pDC carry a functional cGAS/STING axis down-stream of STING, they sense HCMV in a TLR9-dependent manner. However, monocyte-derived cells are triggered by HCMV in a cGAS-dependent manner to mount IFN-I responses. Interestingly, IFI16 cannot compensate cGAS deficiency. Because individuals devoid of MyD88 function do not suffer from enhanced incidence or severity of herpesvirus infections [31,32], it is likely that in the pathogenesis of HCMV infected humans pDC-derived IFN-I do not play a critical role. This study provides evidence that MΦ, which are targets for HCMV infection *in vivo* [73], mount high amounts of MyD88-independent IFN-I and thus may contribute to the protection of MyD88 deficient patients. The moderate STING levels in moDC that might limit IFN-I responses even in the presence of ample cGAMP concentrations imply that moDC are not main providers of MyD88-independent IFN-I responses. So far it remains unclear whether antigen-presenting cells establish cell-cell contact with surrounding tissue cells to transfer cGAMP and thus spread antiviral protection. To address this question the direct cGAMP detection method used in this study might become instrumental for the analysis of cGAMP levels in *ex vivo* isolated infected tissues.

## Materials and Methods

### Cells and viruses

HCMV-GFP was generated on the backbone of the endotheliotropic BAC-cloned TB40/E strain [74–76]. For preparation, the virus was first passaged on HUVEC cells (ATCC: PCS-100-010) and then expanded on MRC-5 cells (ATCC: CCL-171). Viral titers were determined on MRC-5 cells as described previously [19]. VSV-M2 [77] and VSV-eGFP [78] were expanded on BHK-21 cells and titers were determined by plaque formation on Vero cells. MVA-mCherry [79] was propagated and titrated on chicken embryo fibroblasts. Original THP-1 cells and clones carrying a CRISPR/Cas9-mediated knock-out of cGAS or STING [59] as well as of IFI16 were cultured in RPMI1640 containing 10% FCS and 1% sodium pyruvate. Differentiation of THP-1 cells was performed by stimulation with 200 nM phorbol 12-myristate 13-acetate (PMA) for 3 days. One day prior to usage medium was exchanged and cells were cultivated in fresh medium.

IFI16 knock-out THP-1 cells were generated as previously described [80]. An early coding exon of the IFI16 gene was targeted using the following sgRNA target site: 5′-CGGACACCTTACTCCCTTTG-3′. The sgRNA construct was obtained from the sgRNA^KOLIBRY^ library [81]. Following limiting dilution cloning, cell clones harboring all-allelic frame shift mutants were identified using Outknocker [82].

### Primary cell isolation and differentiation

Primary human pDC and monocytes were isolated from buffy coats of healthy blood donors provided by the *Blutbank Springe* (Germany) using ficoll density gradient centrifugation and subsequent magnetic activated cell sorting (Diamond Plasmacytoid Dendritic Cell Isolation Kit, CD14^+^ Cell Isolation Kit; Miltenyi Biotec). Following isolation, 2 x 10^5^ pDC were cultivated for 1 h in 200 μl of 10 ng/ml interleukin 3 containing serum-free DC medium (CellGenix) and were then treated as indicated. moDC, GM-CSF MΦ, and M-CSF MΦ were differentiated from 5 x 10^5^/500 μl monocytes for 5 days in serum-free DC medium enriched with 1000 U/ml GM-CSF (granulocyte macrophage-colony stimulating factor, CellGenix) and 1000 U/ml IL-4 (CellGenix), or 80 U/ml GM-CSF, or 100 ng/ml M-CSF (macrophage-colony stimulating factor, Miltenyi Biotec), respectively.

### Cell stimulation and transfection

Primary human cells were stimulated with MVA-mCherry and VSV-M2 at MOI 1 or HCMV-GFP at MOI 3, except otherwise indicated. THP-1 cells were stimulated with MVA-mCherry and VSV-eGFP at MOI 1. HCMV-GFP infection was performed at MOI 50 in undifferentiated THP-1 cells and at MOI 10 in PMA-matured THP-1 cells. HCMV-GFP infection was enhanced by centrifugation at 300 *g* for 30 min. Cells were stimulated with LPS (100 ng/ml, Sigma-Aldrich), IFN-γ (10 ng/ml, Preprotech), and poly(I:C) (10 μg/ml, InvivoGen) as well as with recombinant IFN-α2b (1000 U/ml, IntronA, MSD Merck Sharp & Dohme AG). 18–24 hpi cell-free supernatant was harvested for ELISA analysis and cells were kept for mRNA, western blot, and flow cytometry analysis. For intracellular cytokine staining, cells were treated with Brefeldin A (BD Bioscience) 6 h prior to intracellular IFN-α staining.

For transfection of cells with synthetic cGAMP (InvivoGen) or siRNA directed against cGAS (SMARTpool: siGENOME MB21D1, GE Healthcare), or control (siGENOME Non-Targeting siRNA Pool #2, GE Healthcare), the Viromer BLUE Kit (Lipocalyx) was used. Final concentrations of 43 nM siRNA were packed in 22 μM Viromer following the manufacturer´s instructions and incubated with the cells for 72 h starting on day 2 after monocyte isolation and differentiation. 48 h post transfection and 1 h prior to stimulation differentiation medium was exchanged. Transfection of 3 μg/ml synthetic cGAMP packed in 22 μM Viromer was performed similarly and cells were analyzed after 12 h by qPCR and supernatants were analyzed by ELISA after 24 h. For inhibition of TLR9 signaling, 7 x 10^4^ pDC were incubated with IRS869 (PTO, 5’-TGCTTGCAAGCTTGCAAGCA-3’) [61] for 1 h and subsequently stimulated with HCMV-GFP at MOI 3 or 5 μg/ml R848 (InvivoGen).

### HPLC-MS/MS quantification of cGAMP

Monocyte-derived cells were lysed 24 hpi with HCMV-GFP by addition of 300 μl of a 2/2/1 [v/v/v] methanol, acetonitrile and water (HPLC-grade, J.T. Baker) mixture containing 25 ng/ml tenofovir (obtained through the NIH AIDS Research and Reference Reagent Program) as an internal standard. Wells were rinsed twice. After incubation for 15 min at 95°C, protein precipitation of lysates was performed at -20°C over night and protein-free lysates were obtained by collection of supernatants after 10 min 20,000 *g* centrifugation. Supernatants were vaporized (Concentrator plus, Eppendorf) and remaining pellets were dissolved in water for mass spectrometry analysis. An HPLC-system (Nexera, Shimadzu), consisting of two HPLC pumps, a temperature controlled autosampler, a degasser, an oven, and a control unit was employed for reversed phase chromatographic separation of cGAMP (2´-5´/3´-5´) and cGAMP (3´-5´/3´-5´) calibrators or sample extracts. A Zorbax eclipse XCB-C18 1.8 μm column (50 x 4.6 mm) kept at 25°C from Agilent was used, connected to a C18 security guard (Phenomenex) and a 2 μm column saver (Supelco). The mobile phases were 3/97 methanol/water [v/v] (A) and 97/3 methanol/water [v/v] (B), each containing 50 mM ammonium acetate and 0.1% acetic acid. The following gradient was applied: 0 to 5 min, 0 to 50% B and 5 to 8 min, 0% B. The flow rate was 400 μl/min. Detection and quantification of cGAMP (2´-5´/3´-5´) was carried out by a tandem mass spectrometer, 5500QTRAP (AB Sciex), equipped with an electrospray ionization source, operating in positive ionization mode. For SRM detection, the following mass transitions were identified for cGAMP (2´-5´/3´-5´): m/z 338.1 [M+2H]^2+^ → 152.0 [M+H]^+^ (quantifier), m/z 338.1 [M+2H]^2+^ → 119.0 or 136.0 [M+H]^+^ (identifier) and for tenofovir: m/z 288.0 [M+H]^+^ → 176.0 [M+H]^+^ (quantifier), m/z 288.0 [M+H]^+^ → 159.1 [M+H]^+^ (identifier). Stock solution of cGAMP (2´-5´/3´-5´) (obtained from Biolog) was prepared in HPLC-grade water. Calibration curves were constructed using seven calibrators ranging from 0.64 to 10,000 nM. Tenofovir was applied as internal standard.

### Flow cytometry analysis

Intracellular IFN-α staining was performed according to the intracellular staining protocol from BD Bioscience using anti-human IFN-α antibody APC (Miltenyi Biotec). Surface marker staining with anti-CD14 V450 (BD Bioscience), anti-CD163 PE, anti-CD206 PE-Cy7, and anti-CD209 APC (BioLegend) was performed for 20 min at 4°C. Data were acquired on a LSRII flow cytometer (BD Biosciences) and analyzed with FlowJo software (Tree Star).

### Immunoblotting

Cells were lysed in SDS sample buffer, denatured at 95°C for 10 min, sonicated for 10 min, and lysates were separated by 10% SDS-PAGE. After transfer to nitrocellulose membranes, membranes were blocked in 5% milk (TBS, 0.1% Tween) before incubation with anti-cGAS (1:1,000; Sigma or 1:1,000; Cell Signaling), anti-STING (1:5,000; R&D Systems or 1:1,000; Cell Signaling), anti-IFI16 (1:2,000; Santa Cruz), anti-P-IRF3 (1:2,000; Cell Signaling), or anti-IRF3 (1:2,000; Cell Signaling) antibodies over night or anti-β-Actin-Peroxidase (1:50,000; Sigma-Aldrich) antibody for 1 h. After 2 h of incubation with secondary goat anti-rabbit-HRP, and goat anti-mouse-HRP (KPL), or goat anti-mouse IgG1-HRP (Southern Biotech) membranes were developed with Amersham ECL Western Blotting Detection Reagent (GE Healthcare). For detection of P-IRF3 SuperSignal West Femto Maximum Sensitivity Substrate (Thermo Scientific) was mixed 1:10 with Amersham ECL Western Blotting Detection Reagent.

### ELISA analysis

Cell-free supernatants were analyzed by using Human IFN-alpha Platinum ELISA (eBioscience), human IFN Beta ELISA kit (PBL), and human IL-12, IL-10 and TNF-α ELISA (BioLegend) according to the manufacturer’s instructions.

### qPCR

RNA extraction (Macherey-Nagel) and cDNA synthesis (Takara) were performed according to the manufacturer’s instructions. 5 ng of cDNA were analyzed by quantitative PCR using SensiFAST SYBR no-ROX Kit (Bioline) in a LightCycler 480 (Roche). All data are presented as relative expression to hypoxanthine phosphoribosyl transferase 1 (HPRT1) mRNA. The corresponding primers were:

HPRT1 forward, 5’-GAACGTCTTGCTCGAGATGTG-3’

HPRT1 reverse, 5’-CCAGCAGGTCAGCAAAGAATT-3’

IFN-α forward, 5’-CGATGGCCTCGCCCTTTGCTTTA-3’

IFN-α reverse, 5’-GGGTCTCAGGGAGATCACAGCCC-3’

IFN-β forward, 5’-TGTGGCAATTGAATGGGAGGCTTGA-3’

IFN-β reverse, 5’-TCAATGCGGCGTCCTCCTTCTG-3’

cGAS forward, 5’-CCCAAGCATGCAAAGGAAGG-3’

cGAS reverse, 5’-ACAATCTTTCCTGCAACATTTCT-3’

STING forward, 5’-CACCTGTGTCCTGGAGTACG-3’

STING reverse, 5’-CATCTGCAGGTTCCTGGTAGG-3’

MxA forward, 5’-ACAGGACCATCGGAATCTTG-3’

MxA reverse, 5’-CCCTTCTTCAGGTGGAACAC-3’

### *In vitro* stimulation of recombinant cGAS

Stimulation of 0.1 μM recombinant human cGAS (residues 155–522) [58] was performed for 2 h at 37°C in the presence or absence of 1 mM ATP and 1 mM GTP in 40 mM TRIS pH 7.5, 100 mM NaCl und 10 mM MgCl_2_. To activate cGAS, 6 μM of a control 50 bp dsDNA (5’-GGATACGTAACAACGCTTATGCATCGCCGCCGCTACATCCCTGAGCTGAC-3’, Eurofins Genomics) was used. Furthermore, purified HCMV containing 6.5 x 10^6^ infectious particles was used with and without DNA digestion by 1 μl Benzonase Nuclease (Merck Millipore) in the presence of 1 mM MgCl_2_ for 30 min at 37°C prior and post heat treatment for 10 min at 95°C. To isolate genomic HCMV DNA, purified HCMV was digested with Benzonase Nuclease for 30 min at 37°C to remove residual DNA that was not packaged into viral particles. Subsequently, digestion by Benzonase Nuclease was inhibited by treatment with 10 mM EDTA and genomic HCMV DNA was isolated using the DNeasy Blood & Tissue Kit (Qiagen). Quantification of isolated HCMV genomes was performed using qPCR analysis in parallel with defined amounts of HCMV BAC DNA (qPCR primers: HCMV forward: GGGTTCTCGTTGCAATCCTC; HCMV reverse: GGAAGGAGGTTAACAGTCAGC). Since this method might also detect fragments of genomic HCMV DNA that contain the amplified region, the calculated amount of HCMV genomes might be slightly overestimated. 10 pM isolated genomic HCMV DNA was used in the *in vitro* stimulation assay of recombinant human cGAS, except otherwise indicated. *In vitro* cGAMP synthesis was stopped by the addition of 300 μl of a 2/2/1 [v/v/v] methanol, acetonitrile and water mixture containing 25 ng/ml tenofovir as an internal standard. Subsequently, samples were prepared as described above to determine cGAMP concentrations by mass spectrometry analysis.

### Statistical analysis

Data were statistically analyzed using the software package GraphPad Prism Version 5.0. Comparisons between monocyte-derived cells as well as stimulation induced responses were analyzed by non-parametric paired Wilcoxon signed rank test. Comparison of pDC with monocyte-derived cells was analyzed by non-parametric unpaired Mann-Whitney test.

### Accession numbers

Human cGAS: UniProt Q8N884; human STING: UniProt Q86WV6; human IFI16: UniProt Q16666; human TLR9: UniProt Q9NR96; human IRF3: UniProt Q14653;

## Supporting Information

S1 Fig Characterization of monocyte-derived cells. (A, B) CD14, CD163, CD206, and CD209 surface marker expression was determined by flow cytometry on monocytes, moDC, GM-CSF MΦ, and M-CSF MΦ. (C) GM-CSF MΦ and M-CSF MΦ were stimulated with LPS + IFN-γ, poly(I:C), or LPS for 24 h and cell-free supernatants were monitored by an ELISA method for production of IL-12, IFN-α, and IL-10. Mean ± SEM of 7–23 (B), 3–19 (C) different donors. ***: p ≤ 0.0006 one-tailed Wilcoxon signed rank test.
(TIF)

S2 Fig Freshly isolated pDC show enhanced cGAS expression. MxA and cGAS mRNA expression were assessed by qPCR in freshly isolated pDC, or pDC cultivated for 24 h in the presence or absence of HCMV. Mean ± SEM of 4 different donors from 2 independent experiments.
(TIF)

S3 Fig Viral DNA that is released upon heat treatment of purified HCMV activates recombinant cGAS. 6.5 x 10^6^ infectious HCMV particles were tested for the presence of cGAMP by a HPLC-MS/MS method. Purified HCMV was DNA digested, subjected to heat treatment at 95°C for 10 minutes, and then mixed with recombinant human cGAS in the presence of ATP and GTP. Mixtures were incubated for 2 h and cGAMP formation was quantified using a HPLC-MS/MS method. Mean ± SEM of 5–7 data points from 2–3 independent experiments. **: p ≤ 0.0025 one-tailed Mann-Whitney test.
(TIF)

S4 Fig CRISPR/Cas9 mediated knock-out of IFI16, cGAS and STING in THP-1 cells. THP-1 WT, and 2 clones of IFI16 Ko (#1, #2) cGAS Ko (#1, #2) and STING Ko (#1, #2) THP-1 cells were analyzed for the expression of IFI16, cGAS and STING by western blot. Actin expression was used as loading control.
(TIF)

S5 Fig Upon HCMV stimulation undifferentiated THP-1 cells mount IFN-β responses in a cGAS-dependent manner. WT, and 2 clones (#1 and #2) of cGAS, IFI16, or STING deficient THP-1 cells were stimulated with (A) HCMV at MOI 50, (B) MVA at MOI 1, or (C) VSV at MOI 1 for 24 h. Cell-free supernatant was tested for IFN-β by an ELISA method (A, B), or cell lysates were tested for IFN-β mRNA expression relative to HPRT1 mRNA expression (C). Lysates of virus infected cells were analyzed for phosphorylated IRF3 (P-IRF3) and IRF3 by western blot (A, B, C). Cell lysates of unstimulated (D) undifferentiated THP-1 cells or (E) THP-1 cells that were differentiated with PMA for 3 days were also tested for P-IRF3 and IRF3 by western blot. Mean ± SEM of 3–5 (A), 3–4 (B), and 5 (C) data points from 3 independent experiments. ns = not significant, *: p ≤ 0.047, **: p ≤ 0.0076 one-tailed Mann-Whitney test.
(TIF)

S6 Fig moDC, GM-CSF MΦ and M-CSF MΦ retain LPS-responsiveness after siRNA-mediated knock-down of cGAS. Untreated or siRNA-mediated cGAS knock-down moDC, GM-CSF MΦ, and M-CSF MΦ were stimulated with LPS and after 24 h of incubation TNF-α levels were determined by an ELISA method. Mean ± SEM of 3 different donors from 2 independent experiments.
(TIF)
