# Supplementary figures and images for: cGAS Senses Human Cytomegalovirus and Induces Type I Interferon Responses in Human Monocyte-Derived Cells
Source: PLoS Pathog. 2016 Apr 8;12(4):e1005546. doi: 10.1371/journal.ppat.1005546 (PMC4825940; doi:10.1371/journal.ppat.1005546)

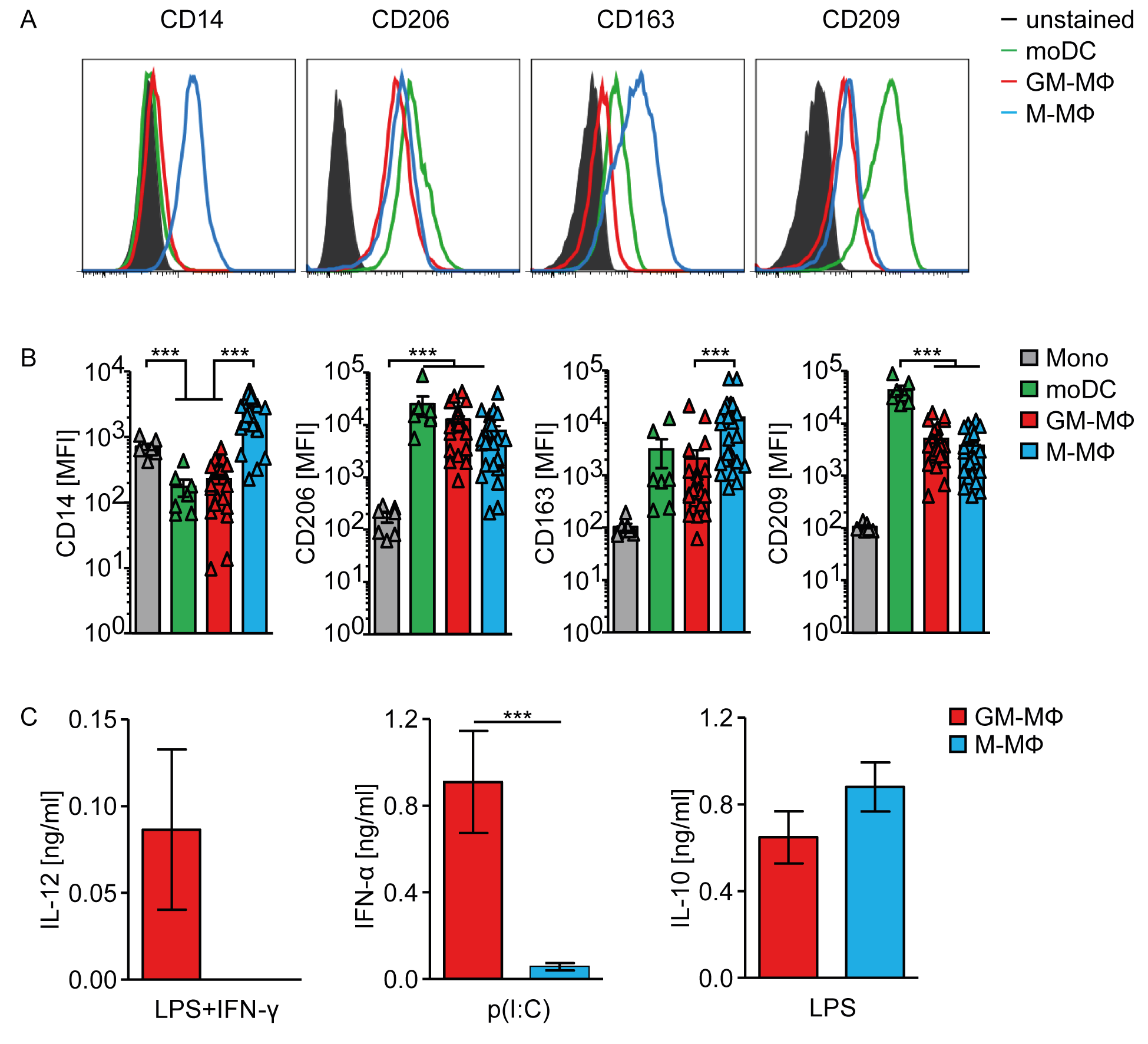

Supplement: S1 Fig — (A, B) CD14, CD163, CD206, and CD209 surface marker expression was determined by flow cytometry on monocytes, moDC, GM-CSF MΦ, and M-CSF MΦ. (C) GM-CSF MΦ and M-CSF MΦ were stimulated with LPS + IFN-γ, poly(I:C), or LPS for 24 h and cell-free supernatants were monitored by an ELISA method for production of IL-12, IFN-α, and IL-10. Mean ± SEM of 7–23 (B), 3–19 (C) different donors. ***: p ≤ 0.0006 one-tailed Wilcoxon signed rank test. (TIF) [file ppat.1005546.s001.tif]

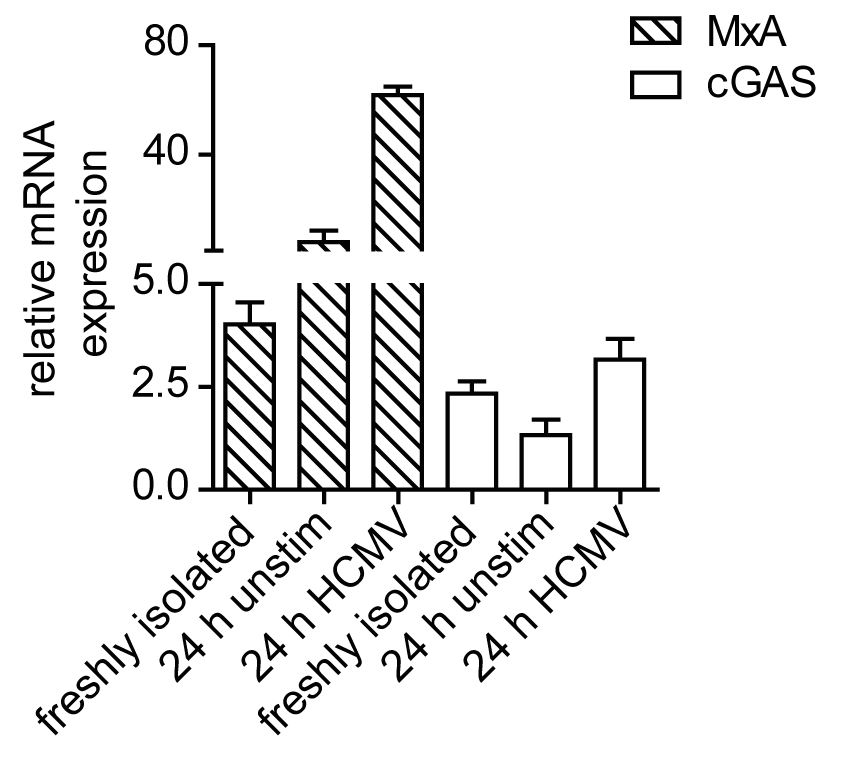

Supplement: S2 Fig — MxA and cGAS mRNA expression were assessed by qPCR in freshly isolated pDC, or pDC cultivated for 24 h in the presence or absence of HCMV. Mean ± SEM of 4 different donors from 2 independent experiments. (TIF) [file ppat.1005546.s002.tif]

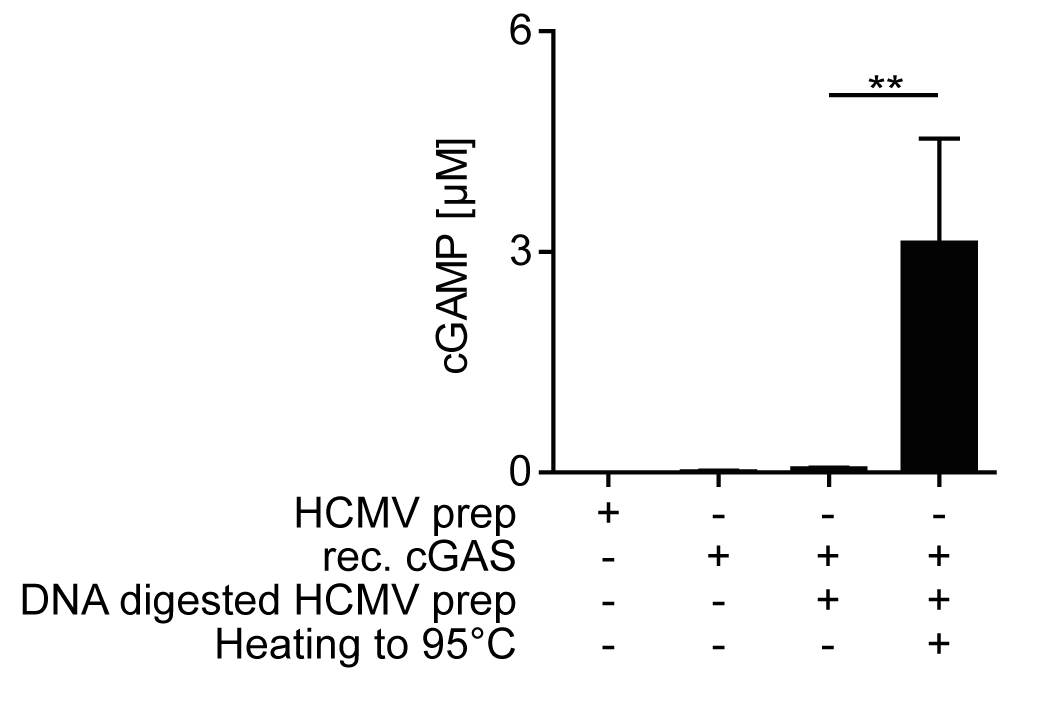

Supplement: S3 Fig — 6.5 x 10^6^ infectious HCMV particles were tested for the presence of cGAMP by a HPLC-MS/MS method. Purified HCMV was DNA digested, subjected to heat treatment at 95°C for 10 minutes, and then mixed with recombinant human cGAS in the presence of ATP and GTP. Mixtures were incubated for 2 h and cGAMP formation was quantified using a HPLC-MS/MS method. Mean ± SEM of 5–7 data points from 2–3 independent experiments. **: p ≤ 0.0025 one-tailed Mann-Whitney test. (TIF) [file ppat.1005546.s003.tif]

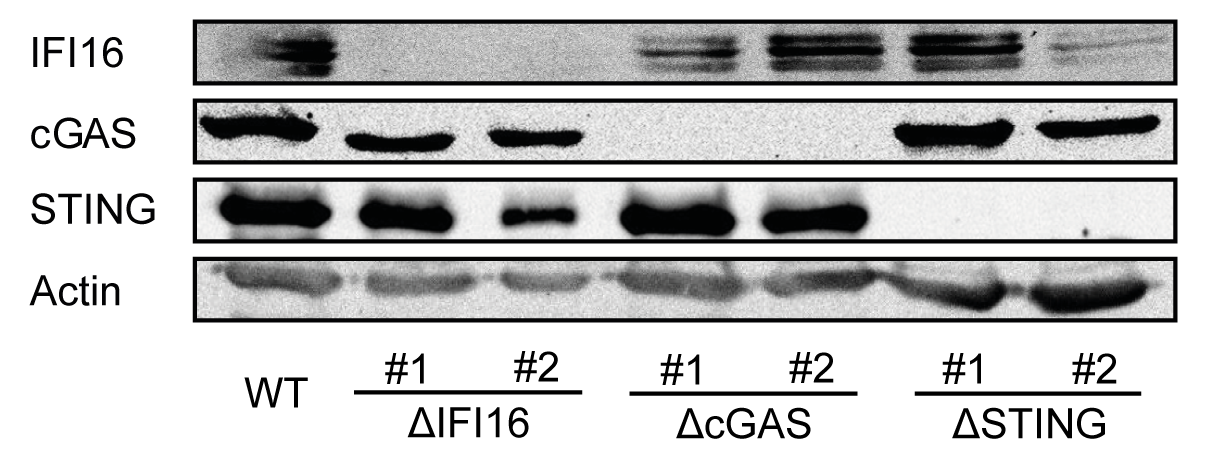

Supplement: S4 Fig — THP-1 WT, and 2 clones of IFI16 Ko (#1, #2) cGAS Ko (#1, #2) and STING Ko (#1, #2) THP-1 cells were analyzed for the expression of IFI16, cGAS and STING by western blot. Actin expression was used as loading control. (TIF) [file ppat.1005546.s004.tif]

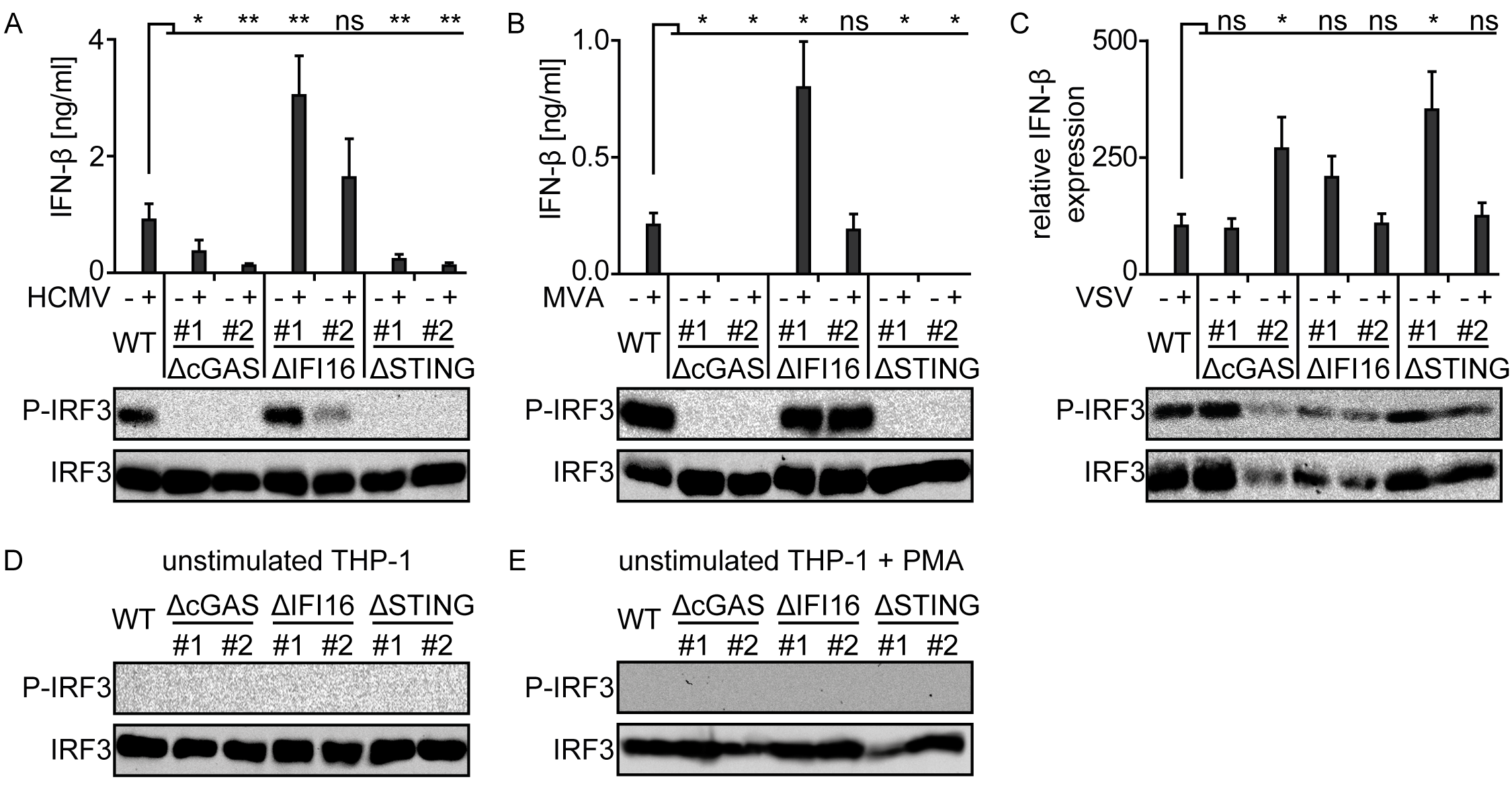

Supplement: S5 Fig — WT, and 2 clones (#1 and #2) of cGAS, IFI16, or STING deficient THP-1 cells were stimulated with (A) HCMV at MOI 50, (B) MVA at MOI 1, or (C) VSV at MOI 1 for 24 h. Cell-free supernatant was tested for IFN-β by an ELISA method (A, B), or cell lysates were tested for IFN-β mRNA expression relative to HPRT1 mRNA expression (C). Lysates of virus infected cells were analyzed for phosphorylated IRF3 (P-IRF3) and IRF3 by western blot (A, B, C). Cell lysates of unstimulated (D) undifferentiated THP-1 cells or (E) THP-1 cells that were differentiated with PMA for 3 days were also tested for P-IRF3 and IRF3 by western blot. Mean ± SEM of 3–5 (A), 3–4 (B), and 5 (C) data points from 3 independent experiments. ns = not significant, *: p ≤ 0.047, **: p ≤ 0.0076 one-tailed Mann-Whitney test. (TIF) [file ppat.1005546.s005.tif]

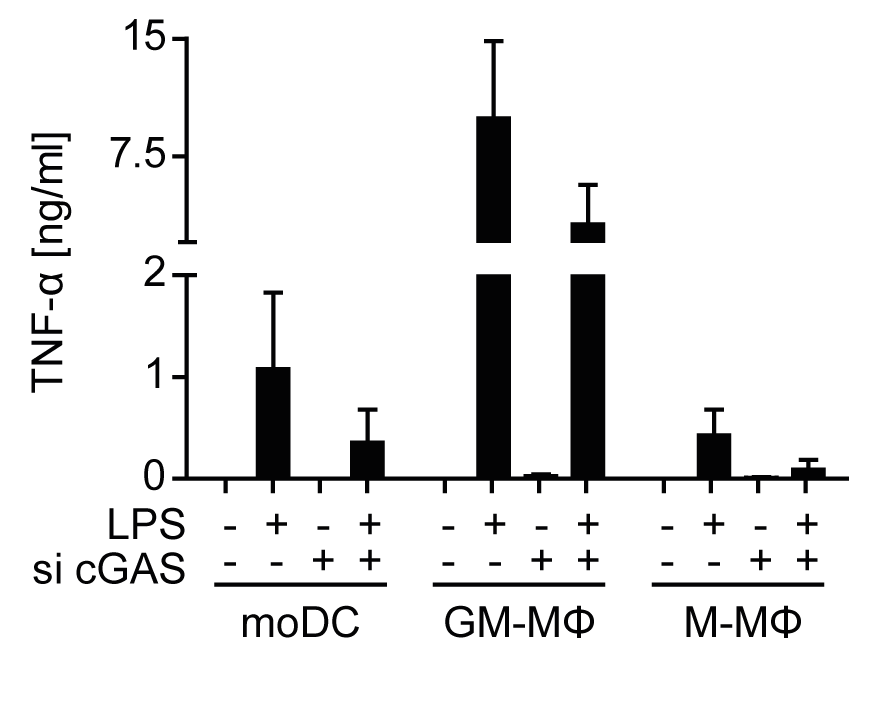

Supplement: S6 Fig — Untreated or siRNA-mediated cGAS knock-down moDC, GM-CSF MΦ, and M-CSF MΦ were stimulated with LPS and after 24 h of incubation TNF-α levels were determined by an ELISA method. Mean ± SEM of 3 different donors from 2 independent experiments. (TIF) [file ppat.1005546.s006.tif]
